# Earwax: A potentially useful medium to identify inborn errors of metabolism?

**DOI:** 10.1002/jmd2.12102

**Published:** 2020-02-14

**Authors:** Stefan Krywawych, Maureen Cleary, Mel McSweeney, Simon Heales

**Affiliations:** ^1^ Department of Chemical Pathology NIHR BRC Great Ormond Street Hospital Foundation Trust London UK; ^2^ Paediatric Laboratory Medicine and Metabolic Medicine Great Ormond Street Hospital Foundation Trust London UK

**Keywords:** acylcarnitines, amino acids, earwax, guanidino metabolites, postmortem

## Abstract

Earwax was investigated as a source to identify patients' different inborn errors of metabolism (IEMs). Acylcarnitines, amino acids, and guanidino metabolites were measured from 28 treated patients with 11 different metabolic disorders including 3 organic acidaemias, 2 fatty acid oxidation defects, 6 amino acid disorders, and 1 peroxisomal abnormality. On the basis of the ratio of different acylcarnitine species relative to free carnitine, isovaleric acidaemia, methylmalonic acidaemia, and long‐chain hydroxyacylCoA dehydrogenase deficiency could be discriminated from the other disorders. For amino acids, neither creatinine nor alternative amino acid proved suitable reference standards against which results could be expressed. However, argininosuccinate and alloisoleucine were present in significantly elevated concentrations in two patients with argininosuccinate lyase deficiency and two patients with branched‐chain ketoacid dehydrogenase deficiency. This study has raised the potential of earwax for investigation of IEMs and may also have role in postmortem investigations. In view of its limited invasiveness, earwax also may have a role as a material to monitor treatment responses and compliance in patients with IEMs.

## 1 INTRODUCTION

Inborn errors of metabolism (IEMs) occur in different pathways, including the metabolism of amino acids, carbohydrates, and fatty acids. A failure to sustain an energy supply dependent upon fat metabolism, on occasions when carbohydrate is limiting, can manifest at any age with a wide clinical presentation including sudden infant death syndrome (SIDS). At autopsy, these infants tend to demonstrate fat deposits in cardiac and skeletal muscle in addition to hepatic tissue. Although molecular testing is playing an increasing role in the diagnosis of inherited metabolic disorders, including the investigation of SIDS, there is still a clear need for biochemical testing, that is, due to factors such as variance of unknown significance.^1^, ^2^, ^3^

Biochemical studies incorporating acylcarnitine analysis evaluate the presence and levels of acylcarnitines species that may be tissue specific and vary with the sampling site due to its proximity to nearby organs.^4^, ^5^, ^6^ Furthermore, for SIDS cases, postmortem delay may influence the plasma acylcarnitine profile due to factors such as tissue leakage of higher concentrations of metabolites into the blood. In our experience, postmortem blood specimens consistently demonstrate raised free carnitine (C0), acetylcarnitine (C2), butyrylcarnitine (C4), hydroxybutyrylcarnitine (OHC4) with variable concentrations of the other longer chain carnitine esters species (unpublished observations). Consequently, this raises the possibility of a missed diagnosis. Alternatively, bile, although possibly more stable, is complicated by the presence of variable quantities of different fatty carnitine esters which can lead to possible over interpretation. Measurement of amino acids and other intermediate metabolites in postmortem tissues is also particularly unreliable due to variable postmortem changes.

Currently, the diagnostic interpretation of patients' bile and/or plasma results, particularly for SIDS cases, is a balanced compromise based upon careful interpretation of results obtained on bile samples in conjunction with those on blood. Analysis of an alternative tissue in which metabolic changes may be less susceptible to the changes outlined above could potentially improve this process. One possibility could be earwax (cerumen) which is located outside the body in a nonaqueous medium and thereby removed from active body processes. Thus, any encapsulated metabolites could therefore potentially be protected. Due to a longer, but as of yet undetermined, turnover time, earwax measurements may represent an average of longer changes in metabolites.

The outer third of the cartilaginous auditory ear canal proximal to the tympanic membrane is involved in the production of earwax.^7^, ^8^, ^9^ There are two genetically determined types of earwax, the dominant wet type and the recessive dry type which contains a lower percentage of lipid material.^10^ It is predominantly comprised of wax, oil, and keratin protein from dead cells accounting for about 60% of the total weight. The lipid oily materials contain saturated and unsaturated fatty acids 12%‐20%, squalene 12%, alcohols, lanosterol, and cholesterol (10% in combination).^11^, ^12^ The latter three are biosynthesized via the 3‐hydroxy‐3‐methyl‐glutarylCoA reductase pathway with the early steps occurring in the cytosol, then in the endoplastic reticulum, and finally within the peroxisome before being secreted by the sebaceous glands. Also earwax has embedded in it the less viscous secretions from the apocrine glands.

Apart from amino acids that have been reported to be present in earwax by semiquantitative techniques as early as in 1953,^13^ the extent of the range of metabolites contained within earwax has not been fully investigated. Furthermore, no information has been published on the amino acid profile in the earwax of patients with disorders in amino acid metabolism or amino acid transport defects which result in deranged plasma amino acid concentrations. Interpretation of plasma or tissue amino acids concentrations collected at postmortem are unreliable as they are subject to similar discrepant changes as described for the acylcarnitines.

To assess the potential value of earwax as a tissue for diagnosing IEMs, we performed a proof of principle study to evaluate whether this material demonstrates the presence of characteristic disease‐specific diagnostic markers.

## 2 METHODS

Following informed consent, 28 patients encompassing 11 inherited conditions were enrolled into this study over a period of 2.5 years (Table 1). Earwax was collected from either the left or right ear onto a cotton bud by a senior member of the nursing staff during an outpatient clinic. To sample the earwax the outer section of the surface, the ear canal was carefully gently wiped with a cotton bud.

**Table 1:** Number of patients and disorder type enrolled for earwax analysis study

Disorder type	Number of patients	Disorder abbreviation
GlurarylCoA dehydrogenase deficiency	n = 3	GA1
IsovalerylCoA dehydrogenase deficiency	n = 2	IVA
Methylmalonic aciduria	n = 1	MMA
Medium chain acylCoA dehydrogenase deficiency	n = 4	MCADDD
Long chain hydroxyacylCoA dehydrogenase deficiency	n = 3	LCHADD
Branched‐chain ketoacid dehydrogenase deficiency	n = 3	BCKDD
Phenylketonuria	n = 6	PKU
Tyrosinaemia Type 1	n = 1	TYR1
Argininosuccinate lyase deficiency	n = 2	ASALD
Argininosuccinate synthase deficiency	n = 2	ASASD
Lysinuric protein intolerance	n = 1	LPI

The earwax was extracted from the cotton bud with 500 μL of 90% methanol and stored at −20°C for analysis.

Appropriate Internal standard were used in all assays. Heavy isotopes intermediate for the acylcarnitines and for the guanidino metabolites including creatinine. Norleucine and amino ethylcysteine were used as internal standards in the analysis and measurement of amino acids.

Amino acids were analyzed as their phenylisothiocyanate derivatives on 30 μL of extract by reverse‐phase HPLC chromatography using an ODS‐bonded silica column (Waters WAT010950) and UV detection at wavelength 254 nm based using a previously reported method.^14^, ^15^

Butylated acylcarnitine esters (60 μL of extract) were analyzed on tandem mass spectrometry by a previously published method with minor modifications.^16^, ^17^

Guanidino metabolites including creatinine measurements (60 μL of extract) were analyzed in their underivatized state by an in‐house tandem mass spectrometry technique.

No analytical interference from the cotton bud could be demonstrated for any of the assays. This was demonstrated by running “blank” assays in the absence of any earwax.

As a further check on the authenticity of glutarylcarnitine, this specific carnitine species was analyzed in the nonbutylated underivatized state by a similar tandem mass spectrometry technique.

As this was a proof‐of‐concept pilot study, no separate unaffected control specimens were collected. Thus, patients with a range of differing inherited defects acted as controls for the patients with the specific inherited defect being evaluated. This was in accordance with the ethical permission granted. Ethical approval was obtained from both the NHS London Hampstead Health Authority and the Great Ormond Street Hospital Ethics Committee.

## 3 RESULTS

Creatinine can be used as a reference compound, particularly in urine. However, for earwax, this proved an unsuitable reference standard against which the results of metabolites of interest could be expressed. Similarly, expressing results against the weight of earwax weight was inappropriate due to the very small amounts of earwax obtained.

In the absence of a suitable reference metabolite and to reduce variability, it was found that the carnitine esters present in the different fatty acid oxidation defects were best expressed as a ratio to the acetylcarnitine species, whereas the results for the organic acid intermediates were most effectively expressed as a ratio to free carnitine. This approach was more discriminating than expression as a percentage of the sum of all the acylcarnitine species.

### 3.1 Acylcarnitine findings in earwax in patients with mitochondrial fatty acid oxidation defects

Earwax from all three patients with LCHADD showed higher ratios of hydroxyhexadecenoylcarnitine, hydroxyhexadecanoylcarnitine, and hydroxyoctadecanoylcarnitine relative to acetylcarnitine than all the other patients studied (Table 2).

**Table 2:** Positive acylcarnitine results in patients with mitochondrial fatty acid oxidation defects and organic acidurias

Ratio (100 x acylcarnitine/acetyl carnitine) in earwax from patients with mitochondrial fatty acid oxidation defects						
Patient	Earwax hydroxyhexadecenoylcarnitine ratio	Earwax hydroxyhexadecanoylcarnitine ratio	Earwax hydroxyoctadecenoylcarnitine ratio	Matching bloodspot hydroxyhexadecenoylcarnitine μmol/L (normal <0.09 μmol/L)^a^	Matching bloodspot hydroxyhexadecanoylcarnitine μmol/L (normal <0.09 μmol/L)^a^	Matching bloodspot hydroxyoctadecenoylcarnitine μmol/L (normal <0.09 μmol/L)^a^
LCHADDa	6.17 ↑ ↑	10.89 ↑ ↑	9.48 ↑ ↑	0.18 ↑	ND	0.14 ↑
LCHADDb	3.51 ↑	7.59 ↑ ↑	2.28 ↑	0.28 ↑	0.14 ↑	0.30 ↑
LCHADDc	2.46 ↑	4.24 ↑	3.22 ↑	0.36 ↑	0.26 ↑	0.25 ↑
Disease control range	0.11‐1.84	0.24‐3.25	0.21‐1.44			

Ratio (100 x acylcarnitine/free carnitine) in earwax from patients with organic acid defects						
Patient	Earwax Isovalerylcarnitine ratio	Matching bloodspot Isovalerylcarnitine μmol/L (normal <0.06 μmol/L)	Earwax propionylcarnitine ratio		Matching plasma methylmalonate μmol/L (normal <1.00 μmol/L)	
IVAa	15.8 ↑ ↑	10.0 ↑ ↑				
IVAb	12.8 ↑ ↑	21.1 ↑ ↑				
MMAa			9.2 ↑		31 ↑	
Disease control range	0.14‐2.72		0.27‐4.06			

a In‐house established reference range.

However, earwax from the four‐well controlled patients with MCADD could not be differentiated from all other patients on the basis of a raised absolute concentration of octanoylcarnitine the characteristic diagnostic marker for this condition or when expressed as a ratio to decanoylcarnitine, acetylcarnitine, or free carnitine concentrations.

### 3.2 Acylcarnitine findings in earwax in patients with organic acid defects

The two patients with IVA had higher earwax isovalerylcarnitine expressed as a ratio to free carnitine than all the other patients studied (Table 2).

The single patient with MMA showed a higher earwax propionylcarnitine to free carnitine ratio than all the other patients studied (Table 2).

Earwax glutarylcarnitine in the three patients with GA1 were similar to all other patients who all showed the presence of glutarylcarnitine as analyzed by the butylated and nonbutylated methods. At the time of sampling, the bloodspot glutarylcarnitine was raised in all three GA1 patients at 1.64, 0.24, and 0.65 μmol/L (normal in house values <0.07 μmol/L).

### 3.3 Amino acid findings in earwax in patients with defects in amino acid metabolism

The results for individual amino acids were generally expressed as the percentage of the total of all the amino acids.

The two patients with ASALD demonstrated higher levels of argininosuccinate in earwax than was seen in the other patients when calculated both as a percentage of total amino acids present and as a ratio to creatinine (Table 3).

**Table 3:** Positive earwax results in patients with amino acid defects

Patient	Earwax arginino‐succinate % of total amino acids	Earwax arginino‐succinate μmol/mmol creatinine			Matching plasma arginino‐succinate μmol/mmol (normal non detectable)	
ASALDa	1.5 ↑	632 ↑			179 ↑	
ASALDb	4.1 ↑	121 ↑			213 ↑	
Disease control range	0‐0.11	0‐27				

	Earwax Allo‐isoleucine % of total amino acids	Earwax Allo‐isoleucine μmol/mmol creatinine	Matching plasma allo‐isoleucine μmol/L (normal <5 μmol/L)^a^	Matching plasma isoleucine μmol/L (normal 26‐100 μmol/L)^a^	Matching plasma leucine μmol/L (normal 65‐220 μmol/L)^a^	Matching plasma valine μmol/L (normal 90‐300 μmol/L)^a^
BCKDDa	0.12	16.6	17↑	98	74	102
BCKDDb	0.11	24.6	152↑↑	146↑	184	338↑
BCKDDc	0.58 ↑	96.7 ↑	223↑↑	210↑	117	319↑
Disease control range	0‐0.41	1.0‐94.7				

a In‐house established reference range.

Although the BCKDD patient presenting with the highest matching plasma alloisoleucine, at the time of collection of earwax, showed the highest earwax alloisoleucine when expressed as percentage of total amino acids concentrations, the other two patients with BCKDD could not be distinguished from the other non BCKDD patients (Table 3).

The six‐well controlled patients with PKU in whom plasma phenylalanine ranged from 113 to 499 mmol/L around the times of sampling did not appear different from the other patients whether the earwax phenylalanine was calculated on the basis of creatinine, percentage of total amino acids, or the phenylalanine/tyrosine ratio.

Similarly, the patient with TYR1 with a plasma tyrosine 418 mmol/L at time of sampling could not be differentiated from other patients when the earwax tyrosine was calculated on the basis of creatinine, percentage of total amino acids, or the tyrosine/phenylalanine ratio.

Generally, the overall amino acid profiles in earwax differ from that seen in plasma, for unlike plasma in which glutamine, alanine, and glycine are present in the highest concentrations, in earwax it is glycine, serine, and alanine.

## 4 DISCUSSION

The specific characteristic acylcarnitine esters, diagnostic for LCHADD, IVA, and MMA were clearly elevated in the earwax from patients with these conditions raising the possibility that this tissue could be suitable for diagnostic purposes. Further larger scale work is now required to confirm this suggestion.

However, earwax acylcarnitines in individuals with MCADD requires further investigation to establish whether earwax octanoylcarnitine and decanoylcarnitine are raised and thereby of diagnostic value in these patients at time of acute decompensation rather than the well patients studied here.

The observed raised propionylcarnitine is not only a marker for MMA but also for propionylCoA carboxylase deficiency. Thus, it is very likely that it would also be raised in the earwax from patients with this condition.

However, for the patients with GA1, on the basis of the observed earwax glutarylcarnitine levels, they could not be discriminated from all the other disorders. Reanalysis of the specimens, specifically for glutarylcarnitine in their underivatized specimen, confirmed the glutarylcarnitine results obtained on the butylated derivatives. Further work is required to determine the source of the glutaric acid and whether it may in any way be associated with the different lipids present in earwax.^11^, ^12^

The overall plasma profile of amino acids concentrations differ from that seen in earwax in which serine is present in the highest concentration and furthermore in addition to other differences, tyrosine is present in higher amounts than phenylalanine, thus the profile in earwax appears closer to that seen in urine.

However, amino acids not normally present such as argininosuccinate were observed to be raised in earwax from the ASALD patients. Similarly, alloisoleucine was clearly strongly raised relative to the amino acids present in one of the BCKDD patients. Possibly the analysis of a larger amount of earwax would offer greater accuracy for measurement of this amino acid and thereby improve discrimination from the non BCKDD patients.

There a degree of interference with alloisoleucine which occurs with the more sensitive method used in this study, and this interference may be more significant with smaller quantities of sample. Although the identity of this compound is unknown, further work is required to improve chromatographic separation. Maple syrup odor has been reported in the earwax of patients with this condition, but this is probably due to the raised concentrations of 2‐oxo‐acids and their decarboxylation products and not the amino acids.^18^

Although no significant abnormalities could be detected in PKU patients and the TYR1 patient to understand the underlying reasons, it may be helpful to study the less well controlled patients in these cases.

Metabolite concentrations in earwax may represent an integral longer term picture resulting from any secretion and absorption processes by cellular transporters involved in the production of earwax. In contrast, plasma represents a short‐term change providing a momentary slice of information solely in that fluid which flows between and bathes the cells and therefore any interpretations between these two mediums are more complex. The characteristic markers of a number of inherited metabolic disorders involving mitochondrial fatty acid oxidation, organic acid metabolism, and amino acid metabolism have been shown to be present in earwax in this study. However, further work is required to determine the extent to which earwax be used as a diagnostic tissue for IEMs and for investigating postmortem cases.

## COMPLIANCE WITH ETHICS GUIDELINES

The authors declare that they have no conflict of interest.

## INFORMED CONSENT

All procedures followed were in accordance with the ethical standards of the responsible committee on human experimentation (institutional and national) and with the Helsinki Declaration of 1975, as revised in 2000.^5^ Informed consent was obtained from all patients for being included in the study.

## AUTHOR CONTRIBUTIONS

S.K.: initial concept, data generation; S.K., S.H.: experimental design, interpretation and writing of manuscript; M.C., M.M.: patient recruitment, reviewing of manuscript; M.C.: clinical interpretation; M.M.: sample collection.
